# Pulmonary Toxicity and the Pathophysiology of Electronic Cigarette, or Vaping Product, Use Associated Lung Injury

**DOI:** 10.3389/fphar.2019.01619

**Published:** 2020-01-14

**Authors:** Hitendra S. Chand, Thivanka Muthumalage, Wasim Maziak, Irfan Rahman

**Affiliations:** ^1^Department of Immunology and Nano-Medicine, Herbert Wertheim College of Medicine, Florida International University, Miami, FL, United States; ^2^Department of Environmental Medicine, University of Rochester Medical Center, Rochester, NY, United States; ^3^Department of Epidemiology, Robert Stempel College of Public Health & Social Work, Florida International University, Miami, FL, United States

**Keywords:** E-cigarettes, vaping, tetrahydrocannabinol (THC), lipoid pneumonia, EVALI

## Abstract

New emerging tobacco products, especially electronic cigarettes (E-Cig) or electronic nicotine delivery systems (ENDS), have gained a huge popularity, particularly in younger populations. The lack of sufficient evidence-based health effect studies has promoted widespread use/abuse with the assumption that E-Cig or ENDS and/or vaping products are safer and less toxic than conventional tobacco smoking. However, the recent escalation in acute lung injuries and their associated fatalities among ENDS or vaping product users has now brought attention to this silent epidemic *via* investigation into the constituents of ENDS/vaping products and their toxic effects on pulmonary health. Accordingly, CDC has declared an “outbreak” of the e-cigarette or vaping product use associated lung injury (EVALI). EVALI is characterized by sterile exogenous pneumonitis like reaction with substantial involvement of innate immune mechanisms. Vitamin-E acetate (VEA) is found in counterfeit cartridges and bronchoalveolar lavage fluid of EVALI patients. Other reports implicated the presence of aromatic/volatile hydrocarbons and oils consisting of medium-chain triglycerides (MCT oil), including terpenes and mineral oil in tetrahydrocannabinol (THC) containing counterfeit vaping products. These compounds are involved in oxidative stress and inflammatory responses in the lung. Here, we provide the perspectives on the recent case reports on EVALI, etiology, and discuss pulmonary toxicity as well as the mechanisms underlying EVALI susceptibility and lung pathophysiology.

## Introduction

The use of electronic cigarettes (E-Cig) or electronic nicotine delivery systems (ENDS) has reached epidemic levels among young adults in the US. Currently, an estimated 10 million US adults and over three million high school adolescents are active ENDS users (including e-juices and pod based systems) with as high as 27.5% of high school students in 2019 (Cullen et al., 2018; Cullen et al., 2019; Gentzke et al., 2019) and 10.5% of middle school students reported current (past month) ENDS use (Wang et al., 2018). As a result, the FDA Commissioner declared in 2018 that ENDS use among youth reached “nothing short of an epidemic proportion of growth” (Printz, 2018).

ENDS products encompass all the E-Cigs, vapes, e-hookahs, vape pens, tank systems, pods, and mods used at high temperatures. Using ENDS products is commonly referred to as vaping. ENDS products work by heating the e-liquid or e-oil provided in cartridges to produce an aerosol/vapor that users inhale into their lungs. The e-liquid or e-oil may contain nicotine, tetrahydrocannabinol (THC) and/or cannabinoid (CBD), and other substances and additives (flavors), that has hugely contributed to the popularity of ENDS use. There are more than 7,000 flavors including fruit or candy essences added to enhance the “experience”.

Effective delivery of nicotine in the form of a vapor utilizes vehicle solvents like propylene glycol (PG) and vegetable glycerin (VG), which are generally regarded safe by the FDA as food additives. Little is known about how ENDS constituents, e.g. PG and VG, affect the respiratory tract and its local immune-inflammatory functions. Emerging evidence indicates that the acute effects of ENDS or vaping products use on the respiratory system are of particular concern (Khan et al., 2018; Sommerfeld et al., 2018; Viswam et al., 2018; Layden et al., 2019; Madison et al., 2019). To date, all 50 US states have reported “a cluster of mysterious pulmonary illnesses” that may be related to E-Cig use with at least 2,291 potential cases and 48 associated fatalities as of December 3, 2019 (Blagev et al., 2019; Chatam-Stephens et al., 2019; Gentzke et al., 2019; Jatlaoui et al., 2019; Kalininskiy et al., 2019; Taylor et al., 2019). In addition, there are at least seven published case reports from 2012 to 2018 describing similar conditions in E-Cig users with no identifiable infectious etiology and the differential diagnosis includes acute lung injury, atypical pneumonitis, eosinophilic or lipoid pneumonia, as reviewed in (Khan et al., 2018; Viswam et al., 2018). Accordingly, CDC has declared an “outbreak” of the e-cigarette or vaping product use associated lung injury or EVALI throughout the United States (Blount et al., 2019a; Moritz et al., 2019; Navon et al., 2019). Nevertheless, the recent EVALI cases from Canada and Barcelona highlight the importance of understanding the pathogenesis of EVALI and the treatment options available outside the United States (Casanova et al., 2019; Landman et al., 2019). This article provides perspectives of e-cig vaping on chemistry, toxicity, and pathological mechanisms on current episodes of EVALI.

### Causes and Symptoms of EVALI

Among EVALI affected subjects where the lung samples were available, abnormal lipid-laden macrophages that are associated with lipoid and other forms of pneumonia were observed. The presenting symptoms include cough, shortness of breath/dyspnea, chest pain, nausea, vomiting/diarrhea, fatigue, fever, and/or weight loss. The fast development of this clinical entity suggests that some subclinical reactions with unknown long term health implications are taking place in the lungs of most ENDS/THC products’ (including wax and dabs) users, with EVALI cases representing the tip of the iceberg. Indeed, sporadic cases of EVALI had been reported in the UK, EU, and elsewhere, despite highly variable public health policies and also in many places there is no clearly identified reporting system for such cases (Rehan et al., 2018; Biondi-Zoccai et al., 2019).

### Pathophysiology of EVALI, and the Presence of Vitamin E Acetate in BALF of EVALI Patients

Most of the subsequent analysis has been geared towards the constituents of E-liquids/E-juice or vaping products like Vitamin-E (alpha-tocopherol) acetate (VEA) which is being implicated as the likely ‘exogenous’ source of lipids in these ENDS-user subjects (Sommerfeld et al., 2018; Viswam et al., 2018; Layden et al., 2019), and perhaps a causal factor because it was detected in the bronchoalveolar lavage fluids (BALF) of several cases with EVALI and ENDS use history (Reidel et al. 2018; Moritz et al., 2019). For e-liquids, VEA (or vitamin A, retinoic acid) is used as an additive to dissolve/dilute (cutting agent) THC oils along with mineral, coconut oil, and triglyceride medium chain oil, and is also used as a thickening agent for other non-THC e-liquids. Thus far, in a nationwide study, VEA, coconut oil, and limonene (terpene) have been identified in 94%, 2%, and 3% of EVALI patient BALF samples, respectively (Blount et al., 2019b). The absence of these compounds in healthy comparators makes VEA the potential causative agent for EVALI. VEA is not harmful when ingested and is found in many foods but when inhaled it can have a protective role against oxidative stress and inflammatory responses (Hybertson et al., 1998; Wang et al., 2002; Hybertson et al., 2005). Further, it acts as a carrier for drug delivery (Shukla et al., 2014), suggesting it may serve as a carrier for THC in the blood and brain of users. It may be possible that when inhaled/vaporized, VEA or its oxidant/radical derivatives may interfere with physiological lung functions by interacting with phospholipids and surfactants of the epithelial lining fluid (Xue et al., 2005; Beattie and Schock, 2009). There is an urgent need for evidence-based studies to assess the role of VEA in the development of EVALI or other ENDS-associated lung conditions. In addition, inhalation toxicology and aerosolization chemical studies are needed to investigate all the other constituents of the e-liquids or the cartridges used by the users/patients.

### Discussion on Toxicity and Biomarkers of EVALI

Recent histopathological reports showed the presence of burnt/blackened lungs, suggesting that aromatic/volatile hydrocarbons, including terpenes (diluent) and oils, are involved in EVALI (Butt et al., 2019; Triantafyllou et al., 2019). There are five components that affect the toxicity of these agents: cutting agents or oils, temperature, flavoring (e.g. vanillin, menthol, camphene, myrcene, pinene, and lemon-limonene), additives, and heavy metals (lead, arsenic, nickel, mercury) based on counterfeit/bootleg cartridges versus legal or medical cartridges. PG/VG, VEA, and medium-chain triglycerides (MCT oil) and mineral oil along with terpenes (camphene, myrcene, pinenes or limonenes used to attract users) are used to dilute THC wax/oil (known as dabs), as well as nicotine in commercial ENDS liquids. Various hydrocarbons (acrolein, 1,3, butadiene, benzene, toluene, and propene) and reactive aldehydes are formed upon heating these compounds to around 500°F. All these cartridges, including CBD containing cartridges are used at around the common voltages (e.g. 3.5 V to 5.5 V) using a specific device. It is also believed that the counterfeit products used cartridges that contained pesticides or pesticides (e.g. myclobutanil) came from extraction of THC oil. It is likely that these products will generate varying degree of particles and particulate matter in microns, and deposit on different sites in the lung.

In contrast to the e-liquid constituents, the lipid derivatives from the ‘endogenous’ source such as the epithelial lining fluid (ELF) and/or lung surfactants and their constituents, i.e. phospholipids, including dipalmitoylphosphatidylcholine (DPPC), could also be associated with the inflammatory responses of innate immune cells of ENDS users. Hence, the airway lipid dysregulation might also be contributing to the ENDS use associated inflammatory responses and perhaps is involved in EVALI as well. Therefore, the rapid and mass spread of ENDS among US young adults, and the seriousness of potentially acute lung reactions (including fatality), calls for a systematic investigation of ENDS use inflammatory responses (Perrine et al., 2019). This would not only culminate in EVALI, but also develop other comorbid conditions involving cardiomyopathies. This problem may be more augmented in those states where recreational THC products are currently banned, where the marketing (street sellers) may be more obvious than in those states where it is acceptable to sell.

Besides the source of the lipid dysregulation, there is an urgent need to establish the biomarkers that correlate with the acute response to ENDS use or EVALI as recommended by recent CDC guidelines (Blount et al., 2019a; Navon et al., 2019). The recent emergence of system biological approaches, such as genomic, epigenetic, metabolomic, lipidomic, proteomic, and transcriptomic, or in combination as a multi-omic approach has shown some promising biomarkers in acute lung injury etiology (Li et al., 2018), and may help in understanding the susceptibility and causative mechanisms of EVALI among ENDS/vaping product including pod/mod users. Nonetheless, substantial research efforts are needed to define better biomarkers in various biological fluids, e.g. plasma/serum, exhaled breath condensate, EBC, and sputum to help diagnose and treat the complex pathophysiology of EVALI (Singh et al., 2019)- ERJ Open. E-cigarettes are highly heterogeneous (with various grades of severity) and evolving in design with different e-liquids with different nicotine levels (including nicotine-free), benzoic acid/benzoate, nicotine salts, and flavoring agents contribute to the variability across e-cigarette products. And the most recent, “fourth generation” ENDS use was reportedly shown to induce transient lung inflammation and gas exchange dysregulation (Chaumont et al., 2019). Though EVALI is significantly involved in subjects vaping adulterated/counterfeit cartridges, the role of those cartridges which are legally available in legalized states cannot be ruled out.

### Disussion on Cellular and Molecular Mechanisms of EVALI

Certain mechanistic approaches can be presented to understand the mechanisms of EVALI. These biochemical, cellular, and molecular changes are known to occur by e-cig vaping. The following sections provide some of the interesting current hallmarks on the observed mechanisms of lung injuries caused by ENDS use.

**Figure 1:**
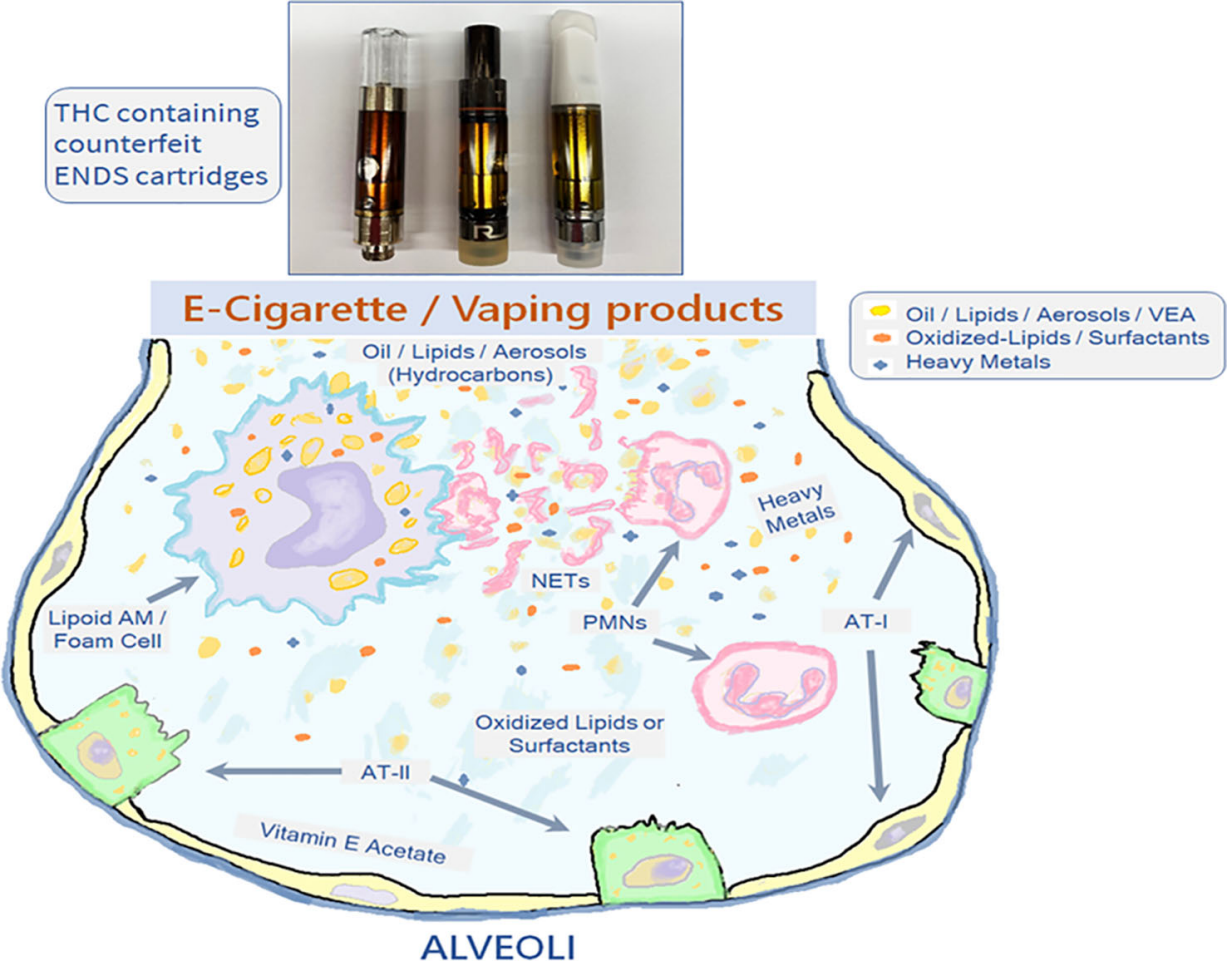
Interaction of ENDS use or vaping generated aerosols/lipids with the airway innate immune cells. The inhaled vapors/aerosols from ENDS use that consists of the hydrocarbons like oil and lipids, vitamin E acetate (VEA) and heavy metals end up in the alveolar regions where active efferocytosis of these aerosolized constituents leads to accumulation of lipid-laden AMs (Lipoid AMs or Foam cells) and the NET-release from the PMNs. The oxidative damage also the leads to the aggregation of oxidative derivatives of the cellular lipids and the surfactants. The AT-II cells secrete inflammatory factors and lung surfactants in the ELF, AMs, alveolar macrophages; AT-1, Alveolar type-1 cells; AT-2, Alveolar type-2 cells; PMNs, polymorphonuclear cells; NETs, neutrophil extracellular traps.

Several physiologic mechanisms, including lung surfactants, mucociliary clearance, and phagocytosis of the inhaled particulates, are paramount in maintaining the airway homeostasis. The airway epithelial cells (AECs), including alveolar type I (AT-I) and type II (AT-II) cells, alveolar macrophages (AMs), and the granulocytes or polymorphonuclear cells (PMNs) are the prominent airway innate immune cells driving these physiological functions and are among the first responders following the ENDS aerosol/vape exposure, as depicted in **Figure 1** AMs are the resident professional phagocytes that ingest and degrade different inhaled irritants, pathogens, and apoptotic cells by ‘‘efferocytosis’’ to help reduce inflammatory responses in the damaged tissues (Ween et al., 2019). Exposure to ENDS and vaping products changes the phenotype and function of AMs and suppresses their efferocytotic activity that helps clear the insult. Hence, reduced efferocytosis will lead to impaired resolution of inflammation. In addition, other cell types including AMs (polarization M1 and M2), PMNs, and AECs are involved in inhaled irritant-induced lung injurious responses.

Several pathophysiological alterations in the lung functions have been associated with E-Cig exposure both in humans and in animal model studies with the reported effects on the airway mucociliary clearance and dysreglated repair mechanisms. In E-Cig users a reduced pulmonary function is often observed that is evaluated by the forced expiratory volume in one second (FEV1) and the ratio of forced expiratory volume to forced vital capacity (FEV/FVC) (Staudt et al., 2018; Meo et al., 2019). Among E-Cig users, a significant increase in MUC4, a membrane-anchored mucin, and an increase in the ratio of secretory mucins MUC5AC to MUC5B has been reported compared to non-smoking participants (Reidel et al., 2018). A cross-sectional study suggested inflammasome complex proteins, caspase-1 and apoptosis-associated speck-like protein containing caspase activation and recruitment domain (ASC), which promote cellular pyroptosis, are elevated in the BAL fluid of E-Cig users (Tsai et al., 2019).

In a limited clinical study, higher serum club cell protein 16 (CC16) levels were strongly correlated in frequent E-Cig users compared to occasional smokers, reflecting epithelial dysfunction/injury in the lungs (Chaumont et al., 2019). In patients with EVALI, there was a higher concentration of serum C-reactive protein (Kalininskiy et al., 2019). Some studies have reported increased inflammatory cell influx in the lung of patients with EVALI (Butt et al., 2019; Layden et al., 2019; Navon et al., 2019; Triantafyllou et al., 2019). In another study, the nasal scrapes from the E-Cig users showed significantly altered expression of early growth response (EGR1), ZBTB16, PIGR, PTGS2, and FKBP5 compared to the occasional E-Cig users with reduced CSF-1, CCL26, and eotaxin-3 levels that are essential for the mucosal host-defense (Martin et al., 2016). A comprehensive biomarker study showed alterations in several inflammatory and oxidative stress mediators in various biological fluids of E-Cig users (Singh et al., 2019). Additional comprehensive, cross-sectional and longitudinal studies are needed to fully establish the toxicity and the pathophysiology of the ENDS product exposure, especially differentially between e-cig nicotine vs THC product vaping on human health.

Clinical data, comprehensive or restrictive, on pulmonary toxicity are lacking; therefore, most of the understanding of the inhalational toxicity of E-Cig aerosol exposure comes from animal studies. In an acute (3 d) exposure mouse model of E-cig exposure, Lerner et al. demonstrated increased BALF IL-6 and CCL2 levels following Blu side-stream aerosol exposure (Lerner et al., 2015). Pod-based e-juices and flavors also induce cellular toxicity with identification of several toxic chemicals (Muthumalage et al., 2019), hence this will render users susceptible to further damage as seen in EVALI cases. Similarly, Wang et al. reported dysregulated lung repair following E-cig exposure (Sundar et al., 2019). Whereas in a long-term exposure (3–6 mo) study using the two strains of mice, higher levels of Angiopoietin-1 and CXCL5 were observed along with lower MMP3 levels, indicating the involvement of the tissue-remodeling pathways (Crotty Alexander et al., 2018). Other rodent model studies have shown potential of DNA damage, adduct formation, and genotoxicity/carcinogenicity of E-Cig and vaping product aerosols (Lee et al., 2018).

Granulocytes, especially PMNs or neutrophils recruited to the injury site rapidly undergo degranulation and set extracellular traps (ETs) to curb the insult. Neutrophil-derived ETs (NETs) are extracellular fibers composed of DNA, histones, and granule-derived proteins such as elastase or myeloperoxidase derived by a process referred to as NETosis (Papayannopoulos, 2018). NETs can trap extracellular irritants and be beneficial during infections to kill invading pathogens; however, aggregated NETs may cause adverse tissue injury to the host. Recently, E-Cig exposure in a mouse model was adversely associated with the increased susceptibility to a bacterial infection due to the disruption in PMNs and dysregulated NETosis (Corriden et al., 2019). Resident monocytes and macrophages are also involved in resolving the NETs-mediated responses (Boe et al., 2015). Moreover, NETs also regulate AM polarization (M1 vs M2) to help mount appropriate immune response (Song et al., 2019).

### Summary and Future Directions

In summary, the following high priority questions should be probed in the ongoing and future research efforts and directions on understanding EVALI pathophysiologies:

1. The most compelling question is to delineate whether the observed EVALI in the reported cases is due to e-cig nicotine/flavor of vaping products in addition to the combined THC oils, or both. Longitudinal studies of ENDS users of nicotine, THC, or the combination of nicotine/THC products can help answer this question.
2. Clinical manifestations following vaping/aerosol exposure are known in all the reported cases, which included inflammation, lipoid- or eosinophilic pneumonia or hypersensitive pneumonitis with a typical ground-glass opacity on the lung CT scans, and the oxygen supplementation along with steroid therapy was able contain the analyzed pathological symptoms. Some patients received prophylactic antibiotics, however, no infection was detected in these patients with EVALI. As EVALI is a manifestation of several components, accordingly the clinical characteristics, diagnosis, and therapeutic interventions are evolving (Cullen et al., 2019; Gentzke et al., 2019; Jatlaoui et al., 2019; Kalininskiy et al., 2019; Chatham-Stephens et al., 2019; Taylor et al., 2019). The recovered patients showed susceptibility to infections, and recent data show that these patients were re-admitted for further treatments. EVALI patients may be carefully monitored post-treatment/discharge for their relapse and recurrence of symptoms due to either remission and/or nature of injuries. Modifying factors include genetic susceptibility, alpha-1 antitrypsin or antioxidant deficiency, among many additional factors. No reports are available on any indicators of what modifying factors account for the pathogenesis and/or patients who recovered after EVALI episodes. Further, the long-term effects of E-cig exposure are yet to be established, partly due to the shorter history of their use as well as the lack of follow-up data on the overall lung health in users.
3. There are no reports on other potentially harmful ingredients besides VEA, which is in fact protective in the lungs as discussed earlier. All the recent reports that implicate VEA to EVALI are based on its detection in the users’ lungs and the e-liquid usage history, but none of the studies provide direct evidence that VEA is the causative agent. All the oils contain VEA and it is very commonly used to dilute the THC oil or is used as a cutting/diluting agent. Other studies have implicated the E-cig flavors with the compromised lung functions, but the etiology so far has prominently pointed towards THC oil containing cartridges.
4. There are several other compounds and ingredients that could be potentially harmful when inhaled as vapors/aerosol and thus, there is an urgent need to analyze the cartridges used and study the pulmonary toxicology, including chemistry, aerosol physico-biological interactions, and pathophysiological mechanisms of counterfeit/bootleg cartridges versus medical dispensary products. Further understanding the chemistry (e.g. chemical compounds, heavy metals, and hydrocarbons), toxicology, and the lung cellular/molecular mechanisms of patients will EVALI could provide much needed information on mechanisms of the disease ultimately leading to regulation of these products, and therapeutic interventions.
5. These studies should be supplemented with thorough chemical analyses specifically for the aerosolization process and its resulting emissions under different conditions, as well as formation of secondary products. It also should include the forensic chemical and the toxicological studies using cell- and animal-based models along with the *ex-vivo* analysis of the clinical samples.
6. Identification of non-invasive biomarkers of ENDS/vaping exposure (plasma/serum, EBC, sputum) and injury progression could provide helpful indicators for early signs of lung damage by these products.
7. Finally, susceptibility factors to EVALI should be researched including genetic and environmental factors, immunocompromising treatments, comorbidities and certain lifestyle choice confounding factors, could also render subjects vulnerable to EVALI and associated pathologies.

Thus, understanding the cause, toxicity and mechanisms of EVALI due to e-cig and/or THC-oil based products vaping will provide the information on regulation of these products and timely therapeutic interventions.

## Author Contributions

All authors wrote and revised/edited the manuscript. HC and IR conceptualized the theme and reviewed the literature. HC and IR wrote and edited the manuscript. TM and WM edited the manuscript.

## Conflict of Interest

The authors declare that the research was conducted in the absence of any commercial or financial relationships that could be construed as a potential conflict of interest.
